# The relationship between of *ACE* I/D and the *MTHFR* C677T polymorphisms in the pathophysiology of type 2 diabetes mellitus in a population of Brazilian obese patients

**DOI:** 10.20945/2359-3997000000005

**Published:** 2018-01-01

**Authors:** Flavio Fontes Pirozzi, Edis Belini, Jessika Viviani Okumura, Mariana Salvarani, Claudia Regina Bonini-Domingos, Milton Artur Ruiz

**Affiliations:** 1 Universidade Estadual Paulista Universidade Estadual Paulista “Júlio de Mesquita Filho” Departamento de Biologia São José do Rio Preto SP Brasil Departamento de Biologia, Universidade Estadual Paulista “Júlio de Mesquita Filho” (Unesp), São José do Rio Preto, SP, Brasil; 2 Associação Portuguesa de Beneficência São José do Rio Preto SP Brasil Associação Portuguesa de Beneficência, São José do Rio Preto, SP, Brasil; 3 União das Faculdades dos Grandes Lagos Faculdade de Medicina São José do Rio Preto SP Brasil Faculdade de Medicina União das Faculdades dos Grandes Lagos (Unilago), São José do Rio Preto, SP, Brasil

**Keywords:** Type 2 diabetes mellitus, obesity, angiotensin-converting enzyme gene, methylenetetrahydrofolate reductase gene, polymorphism

## Abstract

**Objectives:**

This study aimed to evaluate the frequencies of the *angiotensin converting enzyme* (*ACE*) gene insertion/deletion (I/D) and *methylenetetrahydrofolate reductase* (*MTHFR*) gene C677T polymorphisms in obese patients with and without type 2 diabetes mellitus (T2DM).

**Subjects and methods:**

These polymorphisms were analyzed by polymerase chain reaction in 125 patients with obesity, 47 (T2DM) and 78 (Control Group).

**Results:**

No significant difference was found on comparing the T2DM and Control Groups in respect to the genotypic frequencies of the polymorphisms - (II: 13.3% vs. 12.0%; ID: 37.8% vs. 37.3; DD: 48.9% vs. 50.7%; CC: 36.2% vs. 39.0%; CT: 46.8% vs. 49.3%; TT: 17.0% vs. 11.7%), and alleles (I: 32.2% vs. 30.7%; D: 67.8% vs. 69.3%; C: 59.6% vs. 63.6%; T: 40.4% vs. 36.4%) and their synergisms in the pathophysiology of T2DM. On analyzing the T2DM Group, there were no significant differences in the presence of complications. In this population of Brazilian obese patients, no correlation was found between the *ACE* and *MTHFR* polymorphisms in the development of T2DM.

**Conclusion:**

Analyzing only the group with diabetes, there was also no relationship between these polymorphisms and comorbidities.

## INTRODUCTION

Type 2 diabetes mellitus (T2DM) is considered a serious public health problem (1,2). Currently, there are about 415 million diabetics in the world, with more than 14 million in Brazil (3). In addition to the known environmental risk factors such as obesity, there are several genetic changes that are correlated to the pathophysiology of T2DM and its complications (4,5).

As in hypertension, some studies implicate *angiotensin converting enzyme* (*ACE*) gene polymorphisms in the pathogenesis of T2DM, nephropathy and diabetic retinopathy (6,7). The relationship of *MTHFR* polymorphisms associated with hyperhomocysteinemia and changes in the folate cycle is unclear in respect to the development of T2DM with some studies linking these conditions with microvascular complications (8,9).

Epidemiological studies show the relationship of environmental factors in the development of T2DM in the Brazilian population, but few epidemiological studies show the relationship of this metabolic disease with genetic alterations in the same population (10,11), especially in respect to the polymorphisms of the *ACE* and *MTHFR* genes. This study aimed to evaluate the frequency of the insertion/deletion (I/D) polymorphism of the *ACE* gene and the C677T polymorphism of the *MTHFR* gene in a population of obese individuals with and without T2DM. Additionally, the association between these polymorphisms and the occurrence of T2DM and its complications were investigated.

## SUBJECTS AND METHODS

### Subjects

This study enrolled 125 obese adults, 47 with T2DM and 78 nondiabetic patients (Control Group) from June 2015 to November 2015. Inclusion criteria were male and female patients aged 18 to 65 with a body mass index (BMI) greater than or equal to 30 kg/m^2^. The Control Group was matched with respect to gender, BMI and ethnicity (Caucasian and Afro-Brazilian).

Individuals who had two fasting glucose measurements ≥ 126 mg/dL or glycemia levels ≥ 200 mg/dL two hours after ingesting 75g dextrosol or glycated hemoglobin (HbA1c) ≥ 6.5% were considered diabetics according to the criteria of the American Diabetes Association (12). Moreover, patients who were already taking medication to treat diabetes (metformin, sulfonylureas, inhibitor of α-glucosidase, pioglitazone inhibitor, DDP-4, GLP-1 agonists, SGLT-2 inhibitors and insulin) were included in this group.

Microvascular complications, such as diabetic retinopathy (diagnosis by an ophthalmologist), diabetic nephropathy (microalbuminuria, macroalbuminuria or chronic renal failure) and diabetic neuropathy (according to clinical conditions, and physical examination) were also investigated in diabetic patients. Additionally, this study investigated whether the patients had previous diagnoses of hypertension, dyslipidemia or cardiovascular disease (myocardial infarction, stroke and peripheral arterial disease). This research was approved by the Ethics Committee of the Universidade Estadual Paulista “Julio de Mesquita Filho” (UNESP/IBILCE) in São José do Rio Preto, and followed the ethical principles stated in the Declaration of Helsinki. Informed consent was obtained from all participating individuals.

### Sample collection

After fasting for 10 hours, 4 mL of peripheral blood were drawn by venipuncture of the upper limbs and placed into tubes containing 5% EDTA in order to extract DNA from leukocytes (13).

### I/D polymorphism of the ACE gene

The investigation of the I/D polymorphism of 278 bp (rs1799752) in the *ACE1* gene (17q23.3) was carried out by polymerase chain reaction (PCR) using two amplifications with different primers. The first amplification used the ACE1 (sense) 5′CTGGAGACCACTCCCATCCTTTCT3′ and ACE2 (antisense)5′GATGTGGCCATCACATTCGTCAGAT3′ primers; the resulting insertion fragment has 490 bp and the deletion fragment has 190 bp. In this first amplification, there is a preference for the insertion allele to amplify as a deletion allele. Thus, samples homozygous for the deletion allele are submitted to a second amplification to identify heterozygous samples. In this second amplification, the ACE3 (sense) 5′TGGCACGACAGCGCCGCCCACTAC3′ and ACE4 (antisense) 5′TCGCCAGCCCTCCATGCCCATAAT3′ primers are used. The amplified insertion fragment has 355 bp and the deletion fragment is not amplified (14). Both amplifications were analyzed by 2.0% agarose gel electrophoresis under a constant current of 80V for 30 minutes and visualized under ultraviolet (UV) light after staining with ethidium bromide.

### C677T polymorphism of the *MTHFR* gene

The PCR-restriction fragment length polymorphism (RFLP) technique was used to investigate the C677T polymorphism (rs1801133) of the *MTHFR* gene. The MTHFR1 (sense) 5′TGAAGAAGGAGGTGTCTGCGG3′ and MTHFR2 (antisense) 5′AGGACGGTGCGGTGAGAGTG3′ primers were used for amplification generating a 198 bp fragment which was digested using the *HinfI* enzyme (G↓ANTC). The mutation creates a restriction site for the enzyme. The wild homozygous genotype (CC) gives a 198 bp band, the homozygous TT genotype has two bands – one of 175 bp and the other of 23 bp and the heterozygous genotype (CT) gives all three bands (198 bp, 175 bp and 23 bp) (15). The result was analyzed by 4.0% agarose gel electrophoresis under a constant current of 80V for 40 minutes and visualized under UV light after staining with ethidium bromide.

### Statistical analysis

Statistical analyzes were performed using the R program, version 3.2.3 (http://www.rproject.org) and p-values < 0.05 were considered significant. Quantitative variables, tested for normal distribution and homogeneity of variances, are expressed as means ± standard deviation. Mean scores between groups were compared using Student's t test and differences in proportions were evaluated using the Fisher exact test or Pearson chi-square test using the Rcmdr package (16). Allelic and genotypic frequencies and Hardy-Weinberg deviations were evaluated using the SNPassoc package (17). A binary logistic regression model was constructed to evaluate the association of polymorphisms in patients with T2DM in different genetic models of inheritance. The p-value was adjusted for variables using the same logistic regression model using the SNPassoc package (17).

## RESULTS

Table 1 shows the characteristics of the obese, T2DM and nondiabetic Groups (Control Group). The T2DM Group had a mean age of 47.8 ± 8.8 years and the mean age of the Control Group was 41.6 ± 11.5 years (p-value = 0.02). There was no significant differences in relation to the gender or ethnicity.

**Table 1:** Demographic and clinical data of the study participants

Characteristic		T2DM (n = 47)	Controls (n = 78)	p-value
Age (mean ± SD)		47.8 (8.8)	41.6 (11.5)	0.020^c^
Male [n (%)]		17 (36.2)	19 (24.4)	0.157^b^
Female [n (%)]		30 (63.8)	59 (75.6)	
Caucasian [n (%)]		39 (83.0)	62 (79.5)	0.631^b^
Afro-Brazilian [n (%)]		8 (17.0)	16 (20.5)	
Obesity [n (%)]				
	Class I (BMI: 30 – 34.99)	23 (48.9)	43 (55.1)	
	Class II (BMI: 35 – 39.99)	13 (27.7)	24 (30.8)	0.416^b^
	Class III (BMI: ≥ 40)	11 (23.4)	11 (14.1)	
Retinopathy [n (%)]		3 (6.4)	0	< 0.05^a^
Nephropathy [n (%)]		7 (14.8)	0	< 0.05^a^
Neuropathy [n (%)]		7 (14.8)	0	< 0.05^a^
Hypertension [n (%)]		24 (51.1)	30 (38.5)	0.168^b^
Dyslipidemia [n (%)]		25 (53.2)	12 (15.4)	< 0.05^b^
Cardiovascular disease [n (%)]		3 (6.4)	1 (1.3)	0.148^a^

T2DM: type 2 diabetes mellitus; SD: standard deviation

a Fisher exact test;

b Pearson chi-square test or chi-square goodness-of-fit test;

c Student's *t*-test.

There were no statistical differences in the percentages of subjects with Class I, II and III obesity between the T2DM and Control Groups (p-value = 0.416). However, as noted in the general population, both groups had a higher proportion of patients with Class I obesity compared to Class II and III (p-value < 0.01) (18).

There was no significant difference between the two groups in respect to the number of individuals with hypertension and cardiovascular disease. However, the T2DM Group had a higher frequency of dyslipidemia compared to the Control Group. No patients in the Control Group had retinopathy, nephropathy or neuropathy due to other causes, thus the T2DM Group had more patients with these manifestations.

There was no statistically significant differences in the genotypic and allelic frequencies of the polymorphisms of the *ACE* and *MTHFR* genes between the T2DM and Control Groups (Table 2). Additionally, the genotypes of the polymorphisms evaluated showed no predisposition for elevated risk or protection against the development of T2DM.

**Table 2:** Genotypic and allelic frequencies of *ACE* I/D and *MTHFR* C667T polymorphisms in patients with type 2 diabetes mellitus (T2DM) and controls

Genotype/Allele		T2DM	Controls	OR (95% CI)	p-value
*ACE (rs1799752)*		(n = 45)	(n = 75)		
*Genotype*					
	II	6 (13.3)	9 (12.0)	0.87 (0.27-2.77)	
	ID	17 (37.8)	28 (37.3)	0.95 (0.43-2.12)	0.97
	DD	22 (48.9)	38 (50.7)	1.00	
*Allele*					
	I	29 (32.2)	46 (30.7)	0.93 (0.53-1.63)	0.88
	D	61 (67.8)	104 (69.3)		
*MTHFR (rs1801133)*		(n = 47)	(n = 77)		
*Genotype*					
	CC	17 (36.2)	30 (39.0)	1.0	
	CT	22 (46.8)	38 (49.3)	0.98 (0.44-2.16)	0.71
	TT	8 (17.0)	9 (11.7)	0.64 (0.21-1.96)	
*Allele*					
	C	56 (59.6)	98 (63.6)	0.84 (0.49-1.42)	0.59
	T	38 (40.4)	56 (36.4)		

95% CI: 95% confidence interval.

Different models of gene inheritance were evaluated to check any predisposition for elevated risk or protection against T2DM by comparing the two groups. The results are shown in Table 3, and according to the findings, none of the analyzed inheritance models (codominant, dominant, recessive and additive log) have any influence on the development of T2DM in this obese population.

**Table 3:** Analysis of the association of type 2 diabetes mellitus (T2DM) with *ACE* I/D and *MTHFR* C677T gene polymorphisms in different models of inheritance

Model	Genotype	T2DM	Controls	OR (95% IC)	p-value
*ACE (rs1799752)*		n = 45	n = 75		
Codominant	II	6 (13.3)	9 (12.0)	0.87 (0.27-2.77)	0.97
	ID	17 (37.8)	28 (37.3)	0.95 (0.43-2.12)	
	DD	22 (48.9)	38 (50.7)	1.00	
Dominant	DD	22 (48.9)	38 (50.7)	1.00	0.85
	ID-II	23 (51.1)	37 (49.3)	0.93 (0.44-1.95)	
Recessive	II	6 (13.3)	9 (12.0)	1.00	0.83
	DD-ID	39 (86.7)	66 (88.0)	0.89 (0.29-2.68)	
Additive log	-----	-------	------	0.94 (0.55-1.59)	0.81
*MTHFR (rs1801133)*		n = 47	n = 77		
Codominant	CC	17 (36.2)	30 (39.0)	1.0	0.71
	CT	22 (46.8)	38 (49.4)	0.98 (0.44-2.16)	
	TT	8 (17.0)	9 (11.7)	0.64 (0.21-1.96)	
Dominant	CC	17 (36.2)	30 (39.0)	1.0	0.76
	CT-TT	30 (63.8)	47 (61.0)	0.89 (0.42-1.88)	
Recessive	TT	8 (17.0)	9 (11.7)	0.65 (0.23-1.81)	0.41
	CC-TT	39 (83.0)	68 (88.3)	1.00	
Additive log	-----	-------	------	0.84 (0.49-1.43)	0.52

95% CI: 95% confidence interval.

Different combinations of polymorphisms in the T2DM and Control Groups were evaluated in relation to possible synergism between the polymorphisms to check any predisposition for elevated risk or protection against T2DM. Significant differences between groups were not found with any combination of polymorphisms (Table 4).

**Table 4:** Association analysis of type 2 diabetes mellitus (T2DM) using combinations of polymorphisms (*ACE* I/D and *MTHFR* C667T)

Combination	T2DM (n = 45)	Controls (n = 74)	OR (95% IC)	p-value
DD/CC	8 (17.8)	15 (20.3)	0.85 (0.33-2.20)	0.74
DD/CT	12 (26.7)	18 (24.3)	1.13 (0.48-2.64)	0.77
DD/TT	2 (4.4)	5 (6.8)	0.64 (0.12–3.45)	0.63
ID/CC	7 (15.6)	9 (12.2)	1.31 (0.45-3.86)	0.59
ID/CT	7 (15.6)	15 (20.3)	0.72 (0.27-1.94)	0.52
ID/TT	3 (8.8)	3 (3.5)	2.46 (0.51-13.81)	0.35
II/CC	1 (2.2)	5 (6.8)	0.31 (0.03-2.77)	0.41
II/CT	2 (4.5)	3 (4.0)	1.14 (0.18-7.12)	0.89
II/TT	3 (36.2)	1 (39.0)	5.2 (0.53-51.74)	0.15

95% CI: 95% confidence interval.

The number of individuals with T2DM as well as microvascular complications (retinopathy, nephropathy or diabetic neuropathy) or other diseases related to metabolic syndrome such as hypertension, dyslipidemia, and cardiovascular diseases was small and no statistically significant difference was found in relation to polymorphisms of the *ACE* and *MTHFR* genes.

## DISCUSSION

Brazil has one of the highest number of obese and diabetic patients in the world but few epidemiological studies exist, especially related to the association of genetic polymorphisms with this disease and its complications (3,10,11,18). Two groups of obese patients, with and without T2DM, were matched with respect to gender, BMI and ethnicity (Caucasian and Afro-Brazilian). The main objective of this study was to evaluate the frequency of polymorphisms (ACE I/D and MTHFR C677T) and their influence on the pathophysiology of T2DM and microvascular complications in individuals with a BMI ≥ 30 kg/m^2^.

According to the findings of this study, the *ACE* I/D and *MTHFR* C677T polymorphisms are not involved in the development of T2DM in this population. Studies had previously correlated these polymorphisms, especially the I/D polymorphism of the *ACE* gene, with T2DM (4-9) even in Indian women with gestational diabetes (19). These polymorphisms have also been implicated in the development of microvascular complications linked to diabetes (20-22). However, studies in Caucasian populations show no significant relationship with the polymorphism of the *ACE* gene (23). Although the Brazilian population is multiethnic, most of the participants of this study (80.8%) were Caucasians, which supports this finding.

Recent studies point to a synergism between different polymorphisms as the triggering factor for diseases that have a genetic component in their pathogenesis, such as T2DM. Although some studies point to this effect on the development of diabetes, including synergism between the *ACE* I/D and *MTHFR* C677T polymorphisms (24), our study found no significant difference between the patients and controls. Moreover, in the group of patients with T2DM, we did not find significant differences in the analysis of these polymorphisms in relation to microvascular complications and comorbidities such as hypertension, dyslipidemia and cardiovascular disease.

One study reported a higher frequency of the D allele in patients with T2DM and cardiovascular disease and the DD genotype had higher risk for these individuals to present with a cardiovascular event (25). Another study in patients with coronary artery disease, with and without diabetes, showed that the DD genotype is associated with poor coronary collateral circulation, which implies worse ischemic conditioning during acute myocardial infarction (26). The frequencies of the D allele and the DD genotype of the *ACE* gene were higher in both groups pointing to an increased cardiovascular risk probably due to obesity and not T2DM. However, these results should be interpreted with caution, as the numbers of individuals previously diagnosed with cardiovascular disease was very small in both groups.

One study of a cohort of Brazilian subjects did not find any association between the I/D polymorphism of the *ACE* gene and patients with T2DM and metabolic syndrome according to the criteria of the World Health Organization (27), and there are no studies reporting the risk of individuals with this polymorphism developing diabetes. Although there is a Brazilian study that correlates homocysteine levels in patients with and without T2DM to the polymorphism of the *MTHFR* gene (28), there are no studies showing any association of this gene with the risk of developing diabetes in the same population.

Even with the two matched groups, despite the higher age in the T2DM group and more patients with hypertension (not statistically significant) and dyslipidemia (with statistical significance) compared to the Control Group, the number of participants was small in this study and because only patients with obesity were selected, this evaluation did not include overweight patients with metabolic problems.

In conclusion, no association was found between the polymorphisms of the *ACE* and *MTHFR* genes and the development of T2DM in this population. Additionally, no evidence of synergism between the polymorphisms of these two genes and the emergence of diabetes was found. Our group did not find any relationship of polymorphisms of the *ACE* and *MTHFR* genes with microvascular complications and diseases associated to the metabolic syndrome (hypertension, dyslipidemia and cardiovascular diseases) in the group of T2DM patients. This is the first multiethnic Brazilian population study to evaluate polymorphisms of the *ACE* and *MTHFR* genes in obese individuals but our cohort is too small to confirm the data in view of the heterogeneity of the Brazilian population.
